# Which Types of Body-Oriented Interventions Promote Preschoolers’ Social-Emotional Competence? A Systematic Review

**DOI:** 10.3390/healthcare10122413

**Published:** 2022-11-30

**Authors:** Andreia Dias Rodrigues, Ana Cruz-Ferreira, José Marmeleira, Luís Laranjo, Guida Veiga

**Affiliations:** 1 Escola de Saúde e Desenvolvimento Humano, Departamento de Desporto e Saúde, Universidade de Évora, 7004-516 Évora, Portugal; 2 Comprehensive Health Research Centre, University of Évora, 7004-516 Évora, Portugal

**Keywords:** emotional development, social functioning, early childhood education, mind–body, play

## Abstract

There has been a recent increase in body-oriented interventions implemented in educational contexts. Body-oriented interventions are grounded on the body–mind relationship, involving body and movement awareness and expression. In this systematic review of the literature on body-oriented interventions implemented in preschool contexts, we review the scope and quality of the quantitative evidence of each type of body-oriented intervention regarding social-emotional competence. Seven databases were searched for randomized controlled trials (RCTs) and quasi-RCTs. Seven core body-oriented intervention programs were found (e.g., play, relaxation, and psychomotricity). Play programs were the most studied and appear to be the most effective to improve social-emotional competence. Nevertheless, the level of scientific evidence was compromised by the lack of studies with high methodological quality.

## 1. Introduction

Social-emotional competence is crucial for children’s health and well-being. It includes the capacity to understand other’s emotions and feelings, properly regulate and express emotions in different contexts, and positively adapt behavior to form close and secure interpersonal relationships [1,2]. Recent decades of research on children’s social-emotional competence highlight the need to promote these skills since childhood, in particular during the preschool years [3,4], to help children deal with stressors and challenges throughout their lives [1]. Therefore, various intervention programs grounded on specific theoretical frameworks (e.g., cognitive-behavioral theory and behavioral learning theory) have been developed and implemented. Rooted in the body–mind relationship [5,6,7,8], a growing number of body-oriented interventions have been developed in order to improve social-emotional competence. However, compared to other approaches [9,10], research on body-oriented interventions is scarce.

Body-oriented interventions focus on the premise that movement and emotional functioning are associated, and include a broad scope of approaches, such as psychomotricity, relaxation, play and dance [11,12]. Body-oriented interventions put the emphasis on the body as a central arena to the emotional experience, both in a personal and relational approach [12,13,14]. These interventions provide opportunities for awareness of different body signals and states (interoceptive awareness), helping in the identification and association of different body sensations and movements to emotions and mental states [15]. This body awareness is fundamental for a better understanding of own and others’ emotions [15], and therefore for social success.

Results from a prior systematic review [11] revealed moderate evidence that body-oriented intervention programs improve preschoolers’ social-emotional competence (empathy and social interaction), and internalizing and externalizing behaviors. Moreover, results consistently indicated strong scientific evidence for the absence of effects in preschoolers’ ability to delay gratification, as well as on altruistic and aggressive behavior. However, this systematic review focused on the overall effects of the body-oriented intervention, overlooking the effects of each type of body-oriented interventions.

### 1.1. Types of Body-Oriented Interventions and Preschoolers’ Social-Emotional Competence

For example, play-based intervention programs use play as the central mediator. Some programs integrate activities based on specific types of play (e.g., role play, physical activity play). Other programs involve free play, where children have the opportunity to play with different materials and freely engage in different types of play (e.g., role play, constructive play, loose parts play), either alone or in a group.

In dance-based intervention programs, different kinds of dance offer the opportunity to integrate a diversity of movement possibilities. While most interventions focus on children’s spontaneous movements and expression (e.g., creative dance), others focus on specific dance styles (e.g., folk dance).

Relaxation intervention programs aim to obtain the relaxation response, either through attention regulation (e.g., meditation) and/or through muscle tone regulation (e.g., progressive relaxation) [16]. Relaxation intervention programs include different techniques such as breathing techniques, body scanning, stretching, mindfulness, muscular relaxation, or guided imagery.

Psychomotricity focuses on the interactions between movement and body experiences and the mind, integrating the individual cognitive, emotional, social, and physical dimensions. The main mediators are the body in movement, physical activity, and body awareness [14].

Exercise-based intervention programs use different types of physical exercise (e.g., gymnastics, movement games). These programs provide numerous movement opportunities and experiences, allowing children to move their bodies in a structured way, discovering their own body and the environment that surrounds them.

Despite the common conceptual body–mind framework, different mediators can play a distinct role in preschoolers’ social-emotional competence. For example, while relaxation is expected to improve emotion awareness and regulation [16], play-based intervention programs are expected to show better improvements in social competence [17]. Hence, such a detailed analysis has yet to be performed.

### 1.2. Present Study

Contrary to a previous systematic review which followed a general approach of body-oriented interventions [11], the present systematic review focuses on each type of body-oriented intervention. Therefore, the aim of the current study is to examine the strength of scientific evidence regarding the effects of each type of body-oriented interventions on preschoolers’ social-emotional competence. This analysis might help health and educational professionals make evidence-based decisions when implementing body-oriented interventions to promote preschoolers’ social-emotional development in educational contexts. In addition, this systematic review might point out the research gaps that should be addressed in future research focused on body-oriented interventions.

## 2. Methods

The current systematic review followed the Preferred Reporting Items for Systematic Review and Meta-Analysis statement (PRISMA) guidelines [18] and was registered with the International Prospective Register of Systematic Reviews (PROSPERO), on 17 July 2020 (CRD42020172248). A summary of the methods will be presented in this paper. The full details are outlined in Dias Rodrigues and colleagues’ [11] systematic review.

### 2.1. Study Eligibility

The inclusion criteria for the studies were: (1) randomized controlled trials (RCTs) or quasi-RCTs written in English, French and Portuguese, published between 1 January 2000 and 10 October 2020; (2) children’s age ranged between 3 and 7 years old; (3) typical developing children; (4) children enrolled in preschool education; (5) a minimum of one experimental group received body-oriented interventions in the school setting that was carried out by people (as opposed to computers) and; (6) main focus on children’s social-emotional outcomes.

### 2.2. Search Strategy

The following databases were searched on October 10, 2020: CINAHL, ERIC, Portal Regional da BVS, PsycInfo, Pubmed, Scopus, and Web of Science. Additional potentially relevant articles were identified through the manual search of the bibliography of the selected studies’ references. In line with the theoretical framework underlaying body-oriented interventions [12,13,14] and social-emotional competence [1,2], the search terms were related to the different types of body-oriented interventions and other approaches of psychomotricity, combined with social-emotional competence and preschool age terminologies.

### 2.3. Study Selection

In a first phase, two reviewers (A.D.R. and G.V.) independently read all abstracts of the extracted articles and classified them as potentially included or rejected. If there was a disagreement, a third reviewer (J.M.) was consulted. Studies references lists of selected studies were verified as well. In case the search team found some unclear information on those studies, a contact was made with the paper’s corresponding author.

### 2.4. Data Extraction

The extraction of the data was executed by two researchers (A.D.R and G.V.), including authors and study publication year, study characteristics, participants, intervention program implemented, outcomes and measurement instruments, and results. If there were disagreements between the two reviewers, the third reviewer (J.M.) was consulted to come to a conclusion.

### 2.5. Risk of Bias Assessment

Two reviewers (A.D.R. and A.C.-F) independently assessed the methodological quality of the studies using the Physiotherapy Evidence Database (PEDro) scale [19], with the third reviewer (J.M.) consulted to solve any divergences. For each satisfied item of the PEDro scale, one (1) point is obtained. The total score of methodological quality ranges between 1 and 10 (better quality). The following criteria were used to rate method quality: a score of less than 5 indicates “low quality”, and a score of 5, or higher, indicates “high quality” [20,21,22].

### 2.6. Data Synthesis

Outcomes were grouped considering their definition and measurement (see Dias Rodrigues et al. [11]), following Mayo-Wilson [23], Saldanha [24] and colleagues’ suggestions. Afterwards, for each type of body-oriented interventions found, 3 categories were determined: (a) social-emotional outcomes; (b) child’s play; and (c) child’s behaviors.

To measure the studies’ level of scientific evidence, two researchers (A.D.R. and A.C.-F.) used the Best Evidence Synthesis—BES [25], which includes important contributions of meta-analysis and enables the identification of unbiased and meaningful information [25]. The following criteria were used to determine the level of evidence: (a) no evidence—1 low-quality RCT or conflicting results findings; (b) limited evidence—1 high-quality RCT or various low-quality RCTs; (c) moderate evidence—1 high-quality RCT and 1 or more low-quality RCTs included; and (d) strong evidence, multiple high-quality RCTs [26].

## 3. Results

The full details related to the study selection and methodological quality of the selected studies are outlined in Dias Rodrigues and colleagues’ [11] systematic review.

### 3.1. Participant Characteristics

Across all the included studies, participating children were aged between 3 and 7 years old attending preschool education. The participants of nine of the studies were Europeans [27,28,29,30,31,32,33,34,35]. Ethnicity and socioeconomic status data of the participants were not consistently reported by the studies making it difficult to aggregate this information.

The number of participants in each study ranged from 19 [34] to 372 [36]. Only seven studies sampled participants from a single school [27,28,29,34,35,37,38].

### 3.2. Body-Oriented Inttervention Programs Characteristics

We systematized all the body-oriented interventions in seven core programs, namely, play, dance, relaxation, psychomotricity, exercise, and two combined programs. In this context, 10 studies were found focused on play programs [29,30,32,34,35,36,39,40,41,42], 1 study involving a dance intervention program [37], 2 studies with relaxation programs [31,43], 1 study with a psychomotricity program [27], 3 studies regarding exercise programs [33,38,44], 1 study combining play and dance as an intervention program [28], and 1 study combining play and relaxation as an intervention program [45].

Table 1 presents more comprehensive information on the studies.

#### 3.2.1. Play Programs

The duration of play intervention programs ranged from 4 to 60 weeks. Sessions’ frequency ranged from 1 to 5 per week, with sessions’ duration ranging from 30 min to 5 h.

Intervention programs were implemented in the classroom [32,40,42] or in the schoolyard [30,35]. Some of the structured programs were based on manuals and books [34,39,41], and on education programs (e.g., Infant Schools Program in Spain [32]). The activities were child directed [32,39] and/or adult directed [32,36,41,42]. Some interventions used specific materials, such as loose parts [30], animal toys [39,42], puppets [34,39], sandboxes with stones, cars, animal, and fictional characters [40], hula-hoops and balls [35]. In some intervention programs, children were divided into small groups [29,30,34,39,40,41], and other interventions [32,35,41,42] were carried out with the full class of children.

Regarding the types of play observed and/or encouraged through the implementation of play programs, role play [32,34,40,41,42] was the prevalent type. However, physical activity play [29,30,35], constructive play [36], loose parts play [30], and free play with no play type specification [30,35,39] were also included.

#### 3.2.2. Dance Program

The dance intervention program was consisted of two 35 min sessions per week for 8 weeks [37]. The program was based on creative dance and movement and offered structured movement opportunities according to six dance concepts (body parts, movement, space, time, force, and form), which stimulated children to invent multiple and innovative movements according to their personal preferences. Children were divided into small groups of approximately 10 children each.

#### 3.2.3. Relaxation Programs

Relaxation-based interventions’ duration ranged from 1 to 12 weeks. The frequency of sessions ranged from 2 to 3 times per week, with sessions ranging from 11 to 30 min. One of the programs was based on mindfulness activities [43], incorporating children’s literature, music, and movement to teach concepts related to kindness and compassion. The program was provided to children as a part of their standard classroom instruction, during regular school hours. The other program focused on progressive muscle relaxation [31], where the whole class had to listen and follow instructions to tense and release their body muscles.

#### 3.2.4. Psychomotricity Program

The psychomotor intervention program was comprised of two 40 min sessions per week for 8 weeks [27]. The program was based on the pedagogical approach of Psychomotor Education, and the basic principles of the Orff–Schulwerk method [46] of rhythmic education (e.g., clapping and stamping to the beat), developed to promote rhythmic awareness. Role play and noncompetitive team games with a focus on group work were included in each session.

#### 3.2.5. Exercise Programs

Exercise-based intervention programs had a duration of 5 to 14 weeks. The frequency of sessions ranged from 2 to 3 times per week, with sessions’ duration ranging from 30 to 60 min. One of the programs was based on gymnastics educational experiences [44], which was divided into several planned themes aiming to teach basic gymnastic skills (e.g., handstands, running, jumping). The game-based activities program [33] focused on performing movement activities that were listed from simple to difficult. The motor skills intervention program [38] was grounded on the Achievement Goal Theory [47,48,49], focusing on effective instructional pedagogies from the physical education literature and principles, and targeting children’s intrinsic motivation and persistence. This structured movement program focused on critical elements and cue words of motor skills, effective modelling and demonstration, continuous and appropriate feedback, and repetitive cycling of motor skills and tasks. In this study, the intervention program was implemented during the outdoor recess, and children were free to play alone or in a group. The other studies did not report this type of information.

#### 3.2.6. Combined (Play and Dance) Program

The combined (play and dance) intervention program consisted of four 40 min week-sessions for 8 weeks. The program involved folk dance [28] from Baslikesir (Turkey), stimulating specific movements and actions requiring physical coordination. In addition, the program also involved warm-up activities and pre-prepared games based on social physical play (e.g., “The bicycle riding game” and “There is a wave in the sea”) at the beginning and at the end of the sessions.

#### 3.2.7. Combined (Play and Relaxation) Program

The combined (play and relaxation) intervention program consisted of daily 70 min sessions for 1 week [45]. The program combined unstructured free play and mindfulness sessions, with deep-breathing exercises, storytelling, and body scanning. Children had access to loose parts play materials, which have no specific play purpose (e.g., paper boxes, car tires, and tree sticks). Children always had access to the same materials, available in the outdoor playground, which encouraged social play, loose parts play, physical play, and free play.

### 3.3. Effects of Each Body-Oriented Intervention Program on Social-Emotional Outcomes

#### 3.3.1. Play Programs

In social-emotional outcomes, improvements were found in social-emotional competence [36,39], specifically in self-awareness [36], empathy [36,39], emotion recognition [34], and dysfunctional emotional regulation strategies [34]. There were also positive effects in social interaction [35,41], social cooperation [30,35], social independence [35], social assertion [30], and peer relations problems [40]. No differences were found in Theory of Mind [41], emotion identification [34], functional emotion regulation strategies [34], altruism [34,41], and social avoidance [34]. Inconsistent results were present in emotion attribution [34,41], emotion regulation [34,41], self-regulation [29,30,36,39], inhibitory control [42], social competence [30,35,36,39,42], and prosocial behavior [34,40,41].

In children’s play, improvements were found in play, especially in interaction with communication regarding child-directed play, number of groups and interaction with communication regarding teacher-directed play [32]. No differences were found in group composition, number of groups, and group size on child-directed play, and in group size and group composition on teacher-directed play [32].

In children’s behaviors, improvements were reported in general internalizing behaviors [30,35,40], general externalizing behaviors [30,35], behavior problems [35,40], and hyperactivity [30,40]. No positive effects were reported for anxiety/withdrawal [42], and anger/aggression [34,42].

#### 3.3.2. Dance Program

Improvements were reported in all the studied outcomes. More specifically, regarding social-emotional outcomes, improvements were found in social competence. In children’s behaviors, the dance intervention program [37] proved to be effective in decreasing general internalizing behaviors and general externalizing behaviors.

#### 3.3.3. Relaxation Programs

In social-emotional outcomes, improvements were found in social-emotional competence, emotion regulation, social competence, sharing, and prosocial behavior [43]. No differences were found in the delay of gratification [31,43], inhibitory control [31,43], and cognitive flexibility [43].

#### 3.3.4. Psychomotricity Program

No differences were found in the outcome studied—more specifically, in peer acceptance [27].

#### 3.3.5. Exercise Programs

Regarding social-emotional outcomes, exercise programs were found to effectively improve emotion regulation [33], social competence [44], social interaction [44], social cooperation [44], social independence [44], and friendship skills [33]. No differences were found for delay of gratification [38].

#### 3.3.6. Combined (Play and Dance) Program

Improvements were found in the outcome studied—more specifically, in social competence [28].

#### 3.3.7. Combined (Play and Relaxation) Program

The combined play and relaxation program [45] showed improvements in happiness felt after play, play disruption, disconnection, interaction, extent, intensity, and skill. No positive effects were reported regarding emotional expression.

### 3.4. Strength of Evidence

Detailed information regarding the level of scientific evidence of play, dance, relaxation, exercise, and combined programs is presented in Table 2.

#### 3.4.1. Play Programs

Findings regarding social-emotional outcomes indicated the *strong evidence* for the absence of positive effects in altruism, in comparison with 2 active [41] and 1 inactive [34] groups.

We found *moderate evidence* for the improvements in social-emotional competence and empathy compared with inactive groups [36,39]; and social interaction compared with 2 active [41], and 1 inactive group [35], respectively.

*Limited evidence* was observed for improved emotion recognition and dysfunctional emotion regulation strategies compared with inactive group [34], and social cooperation compared with inactive groups [30,35]; and for the nonexistence of positive effects in Theory of Mind compared with two active groups [41], emotion identification, and functional emotion regulation strategies compared with inactive group [34].

There was *no evidence* for improved self-awareness [36], social independence [35], social assertion [30], and peer relations problems [40], all compared with inactive groups. No evidence was observed for the nonexistence of positive effects in social avoidance [34]. The contradictory results revealed no evidence of: emotion attribution comparing with 2 active [41] and 1 inactive group [34]; emotion regulation comparing with 2 active [41] and 1 inactive group [34]; self-regulation in comparison with inactive groups [29,30,36,39]; inhibitory control comparing with 1 active group [42]; social competence comparing with inactive [30,35,36,39] and active groups [42]; and prosocial behavior comparing with 2 active [41] and inactive groups [34,40].

In children’s play, *no evidence* was observed in any of the studied outcomes. More specifically, for the improvements in play, interaction with communication on child-directed play, number of groups, and interaction with communication on teacher-directed play, comparing with an inactive group [32]; and for the absence of positive effects in group composition, number of groups, and group size on child-directed play, as well as for group size and group composition on teacher-directed play, compared with an inactive group [32].

In children’s behaviors, *strong evidence* was observed for the absence of positive effects in anger/aggression compared with active [42] and inactive groups [34].

*Limited evidence* was observed for decreasing general internalizing behaviors in comparison with inactive groups [30,35,40], general externalizing behaviors compared with inactive groups [30,35], behavior problems comparing with inactive groups [35,40], and hyperactivity in comparison with inactive groups [30,40]; and for the nonexistence of positive effects in anxiety/withdrawal comparing with 1 active group [42].

#### 3.4.2. Dance Program

Concerning social-emotional outcomes, *limited evidence* was found for increasing social competence, and in children’s behaviors, for decreasing general internalizing behaviors and externalizing behaviors, compared with one inactive group [37].

#### 3.4.3. Relaxation Programs

*Moderate evidence,* regarding social-emotional outcomes, for the nonexistence of effects in delaying gratification, and inhibitory control compared with inactive groups [31,43].

*No evidence* was observed for improved social-emotional competence, emotion regulation, social competence, sharing, and prosocial behavior, and for the absence of positive effects in cognitive flexibility, compared with an inactive group [43].

#### 3.4.4. Psychomotricity Program

There is *limited evidence* for the absence of positive effects in peer acceptance compared with an inactive group [27].

#### 3.4.5. Exercise Programs

*Limited evidence* was observed for the absence of positive effects in gratification delay compared with an inactive group [38].

*No evidence* was observed for improvements in: emotion regulation and friendship skills compared with an inactive group [33]; social competence, interaction, cooperation, and independence compared with an inactive group [44].

#### 3.4.6. Combined (Play and Dance) Program

There is *no evidence* for positive effects in social competence compared with an inactive group [28].

#### 3.4.7. Combined (Play and Relaxation) Program

There is *limited evidence* for the absence of positive effects in emotion expression [45], and for happiness after, play disconnection, disruption, extent, intensity, interaction, and skill compared with the inactive group [45].

## 4. Discussion

The present study aimed to determine the strength of scientific evidence of play, dance, relaxation, psychomotricity, exercise, and combined programs on the social-emotional competence of preschool age children. While play is the most focused program, with 10 related studies, only 3 RCTs were found regarding exercise, 2 for the relaxation program, and 1 for dance, psychomotricity, and combined programs. The scarce number of studies on dance, relaxation, psychomotricity, exercise, and combined programs did not allow us to find a similarity or standard in the characteristics or methodological quality of each study. As such, in this discussion, we focused only on play programs.

It is important to note that the studies that focused on play are more recent, revealing a recent growth of publications in the last seven years. Play has been long recognized as an essential component of early childhood education by remarkable theorists, such as Vygotsky [50], Piaget [51] or Montessori [52]. This recognition might have contributed to a greater acceptance of play programs in preschools. Such acceptance might also have been reinforced by growing evidence showing the unique contribution of different forms of play for children’s social-emotional development [15,16,53]. Altogether, these factors might facilitate the implementation of play programs in early childhood education compared to other (and less recognized) body-oriented intervention programs.

Play is an instinctive language during childhood, and it also promotes social-emotional development [54,55]. During preschool years, children develop relationships with peers, establish long-term bonds, and build trust and resilience [55,56]. While playing, children enhance their imagination and creativity, engage in pretend play, which allows the development of children’s prosocial behavior, emotion comprehension [34], and emotion regulation [41]. Another common type of play observed in children at this stage is physical activity play (e.g., jumping, running, fighting) [57], which allows children to feel their body and body changes associated with emotional experiences [54]. These are some of the reasons why the importance and benefits of play for children’s development within the school context are increasingly notorious [54].

The different play programs analyzed in this systematic review have distinct features (e.g., frequency and duration of sessions, types of play, materials). As addressed in a previous review [11], it is difficult to determine the best frequency and duration of intervention programs. Despite the emerging consensus that longer durations and frequencies result in better outcomes for children [58,59], as previously observed [11], the findings of the present paper do not support these conclusions. For example, in Loukatari and colleagues’ study [30], the duration of the program was only 4 weeks, with 3 sessions of 30 min per week and significant effects were observed in all the outcomes studied. However, Solomon and colleagues’ study [42], a 60-week program integrated in the school curriculum, showed positive effects in only one of the studied outcomes.

Another reason that may explain the difference in play programs results is the fact that in some studies, assessment was based on teacher’s or parent’s reports. Report-based instruments may be biased, since teachers’ and parents’ perspectives may be different according to the contexts in which children were observed [60,61].

Considering the recent publication of studies focused on play, a higher methodological quality was expected. However, the majority of the studies were of low quality. For example, none of the studies satisfied the criterion of concealed allocation (the person who determined if a subject was eligible for inclusion was unaware of which group the subject would be allocated to), by omission of this information. Again, considering that most play programs studies are recent, this criterion was expected to be satisfied. Another important finding was that in none of the studies, the subject was blind, and only in one study the therapist was blind. The fact that these criteria were not met contributed to the studies’ lower quality.

In fact, strong evidence was only found for play programs, since this type of programs encompasses a greater number of studies (10 studies) with higher methodological quality (4 studies). In the remaining programs, moderate and limited evidence was expected due to the small number of studies that investigated each outcome, even if some of them had higher methodological quality. No evidence was expected in exercise and combined (play and dance) programs considering that each outcome was investigated only by one study of low quality. No evidence due to contradictory findings requires that the outcome is measured by at least two studies. This condition was only verified for play, relaxation, and exercise programs studies. However, contradictory findings were only found for play programs, probably due to the differences between studies characteristics (participants, interventions, and outcome measures) that investigate the same outcome.

In play programs, *strong evidence* that play programs do not improve altruism [34,41], and anger/aggression was found [34,42]. This absence of positive effects on altruism was related to the lack of emotional regulation of the participants, a necessary condition for altruistic behaviors [41]. Regarding the study of Richard and colleagues [34], the lack of positive effects was explained by the low reliability of the post-test assessment of altruism. Concerning the absence of positive results on anger/aggression [34,42], this may be due to the type of play encouraged in these intervention programs. Possibly, role play does not allow children to learn to regulate their aggressive impulses, which a more active form of play does (e.g., exercise play) [15].

There was *moderate evidence* that play programs have positive effects on social-emotional competence [36,39], empathy [36,39], and social interaction [35,41]. Although these programs involved different types of play (i.e., constructive, free, physical and role play, respectively), all of them involved social play, which suggests that, for social-emotional functioning, the social level of play might be more important than the type of play.

*Limited evidence* was observed regarding improvements of play programs on emotion recognition [34], dysfunctional emotion regulation strategies [34], and social cooperation [30,35]. These results were expected since in these play programs, role play and physical play were observed, and it is known both types of play positively relate to emotion recognition and regulation, as well as social competence [62,63,64]. There was also limited evidence for the absence of effects of play programs in Theory of Mind [41], emotion identification [34], and functional emotion regulation strategies [34]. These results contradict what was expected given these programs involved role play, which has been positively related to Theory of Mind [65], emotion identification and regulation [63]. The lack of positive effects on Theory of Mind was attributed to the participants’ poor development of emotional regulation [65]. Concerning children’s behaviors, there was limited evidence for the positive effects of play programs in general externalizing behaviors [30,35], such as behavior problems [35,40], and hyperactivity [30,40]. It is interesting to note that most of these studies involved physical play. Altogether, these findings suggest that physical play interventions are more beneficial for decreasing children’s externalizing behaviors, than role play.

In addition, limited evidence was found for the improvements of play programs in general internalizing behaviors [30,35,40], and for the nonexistence of positive effects in anxiety/withdrawal [42]. The experimental group and active control group were very similar in Solomon and colleagues’ study [42]. Another aspect that may have influenced the results could be the participants’ young age (age range, 3–4), and their socioeconomic status. That is, the greater variability of socioeconomic status may have obscured the benefits of the intervention program since play intervention programs are more effective when implemented to participants with a low socioeconomic level [42].

When contradictory results are found for some outcomes, we conclude that there is a lack of scientific evidence regarding the effects of body-oriented interventions. In this way, *no evidence* was found for emotion attribution and regulation, self-regulation, inhibitory control, social competence, and prosocial behavior. Differences in the programs’ duration and comparison groups [34,41], sessions’ duration and frequency [29,30,36,39], assessment instruments [34,40,41,42], and similarity of the experimental group and active control group [30,35,36,39] might explain these conflictual findings.

In the dance program, *limited evidence* was found for improvements in social competence; and regarding children’s behaviors, in general internalizing behaviors and externalizing behaviors [37]. During this dance intervention program, children were divided into groups, which allowed them to interact with each other, therefore giving them the opportunity to improve their social competence. In addition, dance may give children the possibility of expressing through movement the thoughts and emotions which are difficult to verbally communicate and often expressed through internalizing or externalizing behaviors.

Regarding relaxation programs, there was *moderate evidence* that they do not improve gratification delaying and inhibitory control [31,43]. These results were not expected, since relaxation programs involve controlling the body and/or the mind, by bringing awareness and attention to a particular stimulus (e.g., tension, movement, thought) while inhibiting distractions [16]. Possibly, only older children benefit from relaxation when it comes to the ability to delay gratification and inhibitory control. In fact, studies with older children showed that relaxation programs have a positive effect on these outcomes [66,67]. Intervention program characteristics may explain the absence of positive effects, since the intervention lasted only 1 week with 3 sessions of 11 min, or because one of the comparison groups was an active group.

The psychomotricity program showed limited evidence for absence of positive effects in peer acceptance [27], which can be explained by the low frequency and duration of sessions of the intervention program (2 times per week, for 8 weeks). This result was not expected, since in this intervention program, the type of play encouraged was social role play which has been argued to benefit children’s peer acceptance [68,69].

Concerning exercise programs, there was *limited evidence* for the nonexistence of effects in delay of gratification [38]. These results may suggest that the duration of the intervention program (5 weeks) was not sufficient to improve the ability to delay gratification. It should be noted that the comparison group worsened this ability.

Regarding the combined play and dance program, there was *no evidence* for the positive effects in social competence [28] since the methodological quality of the study was low.

In the combined play and relaxation program, *limited evidence* exists that this type of intervention program does not improve emotion expression [45]. This result was not expected since free play allows the children to express themselves freely, contributing to improved emotional expression [63,70]. Similarly, relaxation programs also provide body and emotional awareness and regulation moments, positively contributing to emotional expression [16,71].

In child’s play, *limited evidence* was observed for the positive effects of combined play and relaxation program in happiness after play, and play disruption, disconnection, interaction, extent, intensity, and skill [45].

### Study Limitations

The limitations of the present review are the following: we excluded all studies that were not RCTs or quasi-RCTs; the validity and reliability of the instruments used in the studies or the adequation of the statistical analysis were not determined; the absence of information about intervention programs characteristics presented in some of the studies; given some interventions were delivered by the classroom teachers and outcome assessments were also completed by them, blinding of assessors or therapists was impossible for some studies, which could lead to a risk of outcome bias; the PEDro scale can lead to a bias of the results, since for one of the items to be satisfied, the study must report that a certain criterion was met; the final limitation is the use of BES, since contradictory results within one type of program of 1 low-quality study led to no evidence of the effects of this type of program in the outcome studied.

Future research should describe in more detail some aspects of the intervention program, such as the materials used, whether the approach is child directed or teacher directed, whether the intervention participants were divided into groups and how many children were included in each group, or if the implementation of the intervention program was in classroom or outdoors. These details might allow us to understand which specificities of the programs bring the most benefits to children.

## 5. Conclusions

This review showed that the majority of the body-oriented intervention programs were play based, although most of them presented a low methodological quality compromising the strength of effects’ evidence of play-based programs on preschoolers’ social-emotional competence. We found strong evidence that play programs do not positively affect preschoolers’ altruism and anger/aggression, and moderate evidence that social-emotional competence, empathy, and social interaction can be promoted with this type of intervention program. These findings suggest that play-based interventions should not be used to manage preschoolers’ anger/aggression nor to promote altruism. However, play-based interventions seem to be beneficial to improve preschooler’s emotional competence, particularly empathy, and social interaction.

In the remaining body-oriented programs, there was only limited and no evidence for the outcomes studied, since the methodological quality of some of the studies was low. Therefore, future studies should consider the following aspects: (1) calculate and report both between-group and within-group effect sizes to compare effect sizes across studies; (2) provide detailed descriptions of the intervention programs to increase the methodological quality, but also to allow researchers and other professionals to replicate them; (3) consider the impact of the intervention dosage, and compare different durations and frequencies of interventions. The need for more studies focused on the effects of dance, relaxation, psychomotricity, exercise, and combined programs is evident.

## Figures and Tables

**Table 1:** Studies characteristics and methodological quality.

Body-Oriented Intervention Program	Source	Study Type/Design	Participants	Intervention Program	Outcomes and Instruments	Findings	PEDro Scale
Play	[32]	RCT Pre–post test	N = 45; 5–6 years EG: *n* = 22 CG: *n* = 23	Dosage: 13 wk, 1 × 300′ per week. EG = group play activities. CG = usual routines.	Sociogrammes ^b^ = play. Mappings—Child-directed play ^d^ = number of groups; group size; group composition; interaction with communication (forms of interaction). Mappings—Teacher-directed play ^d^ = number of groups; group size; group composition; interaction with communication (forms of interaction).	EG = enhanced play. Regarding child-directed play, enhanced interaction with communication; no effects on the remaining outcomes. Regarding teacher-directed play, enhanced number of groups and interaction with communication; no effects on the remaining outcomes. CG = no effects on play. Regarding child-directed play, enhanced solitary play without communication (forms of interaction); no effects on the remaining outcomes. Regarding teacher-directed play, no effects were found on the outcomes.	3
	[36]	RCT Pre–post test	N = 372; 5.1 years EG: *n* = 186 CG: *n* = 186	Dosage: 5 wk, 3 × 90′ per week. EG = group play therapy. CG= usual routines.	Social-emotional Questionnaire ^c^ = self-awareness; self-regulation; social competence; empathy; social-emotional competence.	EG = enhanced all the outcomes. CG = no effects.	3
	[39]	RCT Pre–post test 1-month follow-up	N = 43; 5–6 years EG: *n* = 21 CG: *n* = 22	Dosage: 8 wk, 2 × 30′ per week. EG = child-centered group play therapy. CG = usual routines.	Social-Emotional Assets and Resilience Scale—Parent ^c^ = self-regulation; social competence; empathy; social-emotional competence. Social-Emotional Assets and Resilience Scale—Teacher ^e^ = social-emotional competence.	EG = enhanced all the outcomes except self-regulation and social-emotional competence (reported by teachers). CG = no effects.	5
	[41]	RCT Pre–post test	N = 86; 4–5 years EG: *n* = 31 CG 1: *n* = 29 CG 2: *n* = 26	Dosage: 8 wk, 3 × 30′ per week. EG = pretend play games. CG 1 = block building activities. CG 2 = story time reading.	Theory of Mind Scale ^a^ = Theory of Mind. Sticker “Dictator Game” ^a^ = altruism. Berkeley Puppet Interview Method ^a^ = emotion attribution. Live hurt protocols—Adapted ^d^ = emotion regulation; prosocial behavior. Social Interaction Observation System ^d^ = social interaction.	EG = enhanced emotion regulation, and social interaction; reduced emotion attribution; no effects on the remaining outcomes. CG 1 = no effects. CG 2 = no effects.	7
	[42]	RCT Pre- mid- and post test (0, 8 m, 15 m)	N = 256; 3–4 years EG = 148 CG = 108	Dosage: 60 wk, 5 × integrated on preschool curriculum. EG = play-based with a teacher-directed approach. CG = play-based with a child-centered approach.	Day/Night Task ^a^ = inhibitory control. Head-To-Toes Task ^a^ = inhibitory control. Social Competence Behavior Evaluation—Preschool Edition ^e^ = social competence; anger/aggression; anxiety/withdrawal.	EG = participants with high levels of initial hyperactivity/inattention, increased inhibitory control (Head-To-Toes Task); no effects on the remaining outcomes. CG = no effects.	6
	[29]	Quasi-RCT Pre–post test	N = 30; 5 years EG: *n* = 15 CG: *n* = 15	Dosage: 12 wk, 5 × 45–60′ per week. EG = embedded learning-based movement education. CG = usual routines.	Child Behavior Rating Scale ^d^ = self-regulation.	EG = enhanced the outcome. CG = enhanced the outcome.	4
	[30]	RCT Pre–post test	N = 60; 5–6 years EG: *n* = 30 CG: *n* = 30	Dosage: 4 wk, 3 × 30′ per week. EG = structured playful activities. CG = usual routines.	Social Skills Rating System ^e^ = social cooperation; social assertion; self-regulation; externalizing behaviors; internalizing behaviors; hyperactivity; social competence.	EG = enhanced all the outcomes. CG = no effects.	3
	[34]	RCT Pre–post test	N = 19; 5.7 years EG: *n* = 9 CG: *n* = 10	Dosage: 11 wk, 1 × 60′ per week. EG = pretend play based. CG = usual routines.	Emotional Vocabulary Test ^a^ = emotion recognition. Perceptual Identification of Emotional Facial Expressions Task ^a^ = emotion identification; emotion attribution (anger, disgust, fear, and sadness identification). Comprehension of Causes of Emotions Task ^a^ = emotion attribution. Contextual Task ^a^ = emotion attribution. Structured Interview about strategies for regulating negative emotions ^b^ = functional emotion regulation strategies; dysfunctional emotion regulation strategies. Emotion Regulation Checklist ^c^ = emotion regulation. Altruistic Initiatives Task ^a^ = altruism. Challenging Situation Task—Revised ^a^ = prosocial behavior; anger/aggression; social avoidance.	EG = enhanced emotion recognition, attribution and attribution on anger, disgust, fear, and sadness identification, and dysfunctional emotion regulation strategies; no effects on the remaining outcomes. CG = enhanced emotion attribution (Perceptual Identification of Emotional Facial Expressions Task), and altruism; no effects on the remaining outcomes.	5
	[40]	Quasi-RCT Pre–post test	N = 76; 4–7 years EG: *n* = 45 CG: *n* = 31	Dosage: 6 wk, 1 × 60′ per week. EG = symbolic sand play. CG = usual routines.	Strengths and Difficulties Questionnaire ^e^ = internalizing behaviors; behavior problems; hyperactivity; peer relations problems; prosocial behavior.	EG = enhanced all the outcomes. CG = enhanced hyperactivity; no effects on the remaining outcomes.	3
	[35]	Quasi-RCT Pre–post test	N = 40; 4–6 years EG: *n* = 20 CG: *n* = 20	Dosage: 4 wk, 2 × 45′ per week. EG = structured play activities. CG = usual routines.	Preschool Kindergarten Behavior Scale ^e^ = social cooperation; social independence; social interaction; social competence; externalizing behaviors; internalizing behaviors; behavior problems.	EG = enhanced all the outcomes. CG = enhanced all the outcomes except social independence, and externalizing behaviors.	3
Dance	[37]	RCT Pre–post test	N = 40; 3–5 years EG: *n* = 21 CG: *n* = 19	Dosage: 8 wk, 2 × 35′ per week. EG = creative dance/movement. CG = usual routines.	Social Competence Behavior Evaluation—Preschool Edition ^c,e^ = social competence; internalizing behavior problems; externalizing behavior problems.	EG = enhanced all the outcomes. CG = no effects.	6
Relaxation	[43]	RCT Pre–post test	N = 68; 4.67 years EG: *n* = 30 CG: *n* = 38	Dosage: 12 wk, 2 × 20–30′ per week. EG = mindfulness-based activities. CG = usual routines.	Teacher Social Competence Scale ^e^ = prosocial behavior; emotion regulation; social competence. School grades records ^e^ = social-emotional competence. Sharing Task ^a^ = sharing. Delay of Gratification Task ^a^ = delay of gratification. Dimensional Change Card Sort Task ^a^ = cognitive flexibility. Flanker Task ^a^ = inhibitory control.	EG = enhanced all the outcomes except delay of gratification, cognitive flexibility and inhibitory control. CG = enhanced prosocial behavior, emotion regulation, social competence, and decreased sharing; no effects on the remaining outcomes.	4
	[31]	RCT Pre–post test	N = 101; 6.24 years EG: *n* = 33 CG 1: *n* = 30 CG 2: *n* = 38	Dosage: 1 wk, 3 × 11′ per week. EG = progressive muscle relaxation. CG 1 = attention training technique. CG 2 = usual routines.	Marshmallow Test ^a^ = delay of gratification. Day/Night Task ^a^ = inhibitory control.	EG = no effects. CG 1 = enhanced all the outcomes. CG 2 = no effects.	6
Psychomotricity	[27]	RCT Pre–post test	N = 29; 3–5 years EG: *n* = 14 CG: *n* = 15	Dosage: 8 wk, 2 × 40′ per week. EG= psychomotricity. CG = usual routines.	Pictorial Scale of Perceived Competence and Social Acceptance for Young Children ^b^ = peer acceptance.	EG = no effects. CG = no effects.	5
Exercise	[44]	RCT Pre–post test	N = 60; 3–6 years EG: *n* = 30 CG: *n*= 30	Dosage: 12 wk, 2 × 60′ per week. EG = gymnastics. CG = usual routines.	Preschool and Kindergarten Behavior Scale ^c^ = social cooperation; social interaction; social independence; social competence.	EG = enhanced all the outcomes. CG = no effects.	4
	[33]	RCT Pre–post test	N = 42; 4 years EG: *n* = 21 CG: *n* = 21	Dosage: 14 wk, 2 × 30–50′ per week. EG = game-based activities. CG = usual routines.	Preschool Social Skills Rating Scale ^c,e^ = friendship skills; emotion regulation.	EG = enhanced all the outcomes (better than CG). CG = enhanced all the outcomes.	4
	[38]	RCT Pre–post test	N = 113; 4.01 years EG: *n* = 68 CG: *n* = 45	Dosage: 5 wk, 3 × 40′ per week. EG = motor skills intervention. CG = usual routines.	Delay of Gratification Snack Task—Preschool Self-Regulation Assessment ^a^ = delay of gratification.	EG = no effects. CG = reduced the outcome.	5
Combined (play and dance)	[28]	Quasi-RCT Pre–post test	N = 40; 5–6 years EG: *n* = 20 CG: *n* = 20	Dosage: 8 wk, 4 × 40′ per week. EG = folk dance. CG = usual routines.	Social Adjustment and Skills Scale ^e^ = social competence.	EG = enhanced the outcome. CG = no effects.	4
Combined (play and relaxation)	[45]	Quasi-RCT Pre–post test	N = 42; 4–6 years EG: *n* = 20 CG: *n* = 22	Dosage: 1 wk, 5 × 70′ per week. EG = loose parts play and mindfulness. CG = usual routines.	Smiley Face Likert Scale ^b^ = happiness after play. Children’s Emotional Manifestation Scale ^d^ = emotion expression. Penn Interactive Peer Play Scale ^d^ = play disruption; play disconnection; play interaction. Test of Playfulness Scale ^d^ = play extent; play intensity; play skill.	EG = enhanced all the outcomes except emotion expression. CG = enhanced play extent, intensity, and skill; no effects on the remaining outcomes.	5

Note: ^a^ Instrument measure applied to children; ^b^ child-reported measure; ^c^ parent-reported measure; ^d^ researcher-reported measure; ^e^ teacher-reported measure; EG = experimental group; CG = control group.

**Table 2:** Strength of the evidence on the effects of each body-oriented intervention program.

Body-Oriented Intervention Program	Strength of Evidence	Outcomes	Source	Effects	Methodological Quality
Play	Moderate Evidence	Social-emotional competence	[36]	+	3
			[39]	+	5
	No evidence	Self-awareness	[36]	+	3
	Moderate evidence	Empathy	[36]	+	3
			[39]	+	5
	Limited evidence	Theory of Mind	[41]	−	7
	Limited evidence	Emotion identification	[34]	−	5
	Limited evidence	Emotion recognition	[34]	+	5
	No evidence	Emotion attribution	[41]	−	7
			[34]	+	5
			[34]	+	5
			[34]	−	5
	No evidence	Emotion regulation	[41]	+	7
			[34]	−	5
	Limited evidence	Functional emotion regulation strategies	[34]	−	5
	Limited evidence	Dysfunctional emotion regulation strategies	[34]	+	5
	No evidence	Self-regulation	[36]	+	3
			[39]	−	5
			[29]	+	4
			[30]	+	3
	No evidence	Inhibitory control	[42]	+	6
			[42]	−	6
	No evidence	Social competence	[36]	+	3
			[36]	+	3
			[39]	+	5
			[42]	−	6
			[30]	+	3
			[35]	+	3
	Moderate evidence	Social interaction	[41]	+	7
			[35]	+	3
	Limited evidence	Social cooperation	[30]	+	3
			[35]	+	3
	No evidence	Social independence	[35]	+	3
	No evidence	Social assertion	[30]	+	3
	Strong evidence	Altruism	[41]	−	7
			[34]	−	5
	No evidence	Social avoidance	[34]	−	5
	No evidence	Peer relations problems	[40]	+	3
	No evidence	Prosocial behavior	[41]	−	7
			[34]	−	5
			[40]	+	3
	No evidence	Play	[32]	+	3
	No evidence	Interaction with communication on child-directed play	[32]	+	3
	No evidence	Number of groups on teacher-directed play	[32]	+	3
	No evidence	Interaction with communication on teacher-directed play	[32]	+	3
	No evidence	Group composition on child-directed play	[32]	−	3
	No evidence	Number of groups on child-directed play	[32]	−	3
	No evidence	Group size on child-directed play	[32]	−	3
	No evidence	Group size on teacher-directed play	[32]	−	3
	No evidence	Group composition on teacher-directed play	[32]	−	3
	Limited evidence	General internalizing behaviors	[30]	+	3
			[40]	+	3
			[35]	+	3
	Limited evidence	Anxiety/withdrawal	[42]	−	6
	Limited evidence	General externalizing behaviors	[30]	+	3
			[35]	+	3
	Strong evidence	Anger/aggression	[42]	−	6
			[34]	−	5
	Limited evidence	Behavior problems	[40]	+	3
			[35]	+	3
	Limited evidence	Hyperactivity	[30]	+	3
			[40]	+	3
Dance	Limited evidence	General internalizing behaviours	[37]	+	6
	Limited evidence	General externalizing behaviours	[37]	+	6
	Limited evidence	Social competence	[37]	+	6
Relaxation	No evidence	Social-emotional competence	[43]	+	4
	No evidence	Emotion regulation	[43]	+	4
	Moderate evidence	Delay of gratification	[31]	−	6
			[43]	−	4
	Moderate evidence	Inhibitory control	[31]	−	6
			[43]	−	4
	No evidence	Cognitive flexibility	[43]	−	4
	No evidence	Social competence	[43]	+	4
	No evidence	Sharing	[43]	+	4
	No evidence	Prosocial behavior	[43]	+	4
Psychomotricity	Limited evidence	Peer acceptance	[27]	−	5
Exercise	No evidence	Social competence	[44]	+	4
	No evidence	Social interaction	[44]	+	4
	No evidence	Social cooperation	[44]	+	4
	No evidence	Social independence	[44]	+	4
	No evidence	Emotion regulation	[33]	+	4
	No evidence	Friendship skills	[33]	+	4
	Limited evidence	Delay of gratification	[38]	−	5
Combined (play and dance)	No evidence	Social competence	[28]	+	4
Combined (play and relaxation)	Limited evidence	Play disruption	[45]	+	5
	Limited evidence	Play disconnection	[45]	+	5
	Limited evidence	Play interaction	[45]	+	5
	Limited evidence	Happiness after play	[45]	+	5
	Limited evidence	Play extent	[45]	+	5
	Limited evidence	Play intensity	[45]	+	5
	Limited evidence	Play skill	[45]	+	5
	Limited evidence	Emotion expression	[45]	−	5

Note: + positive results, experimental group(s) progressed compared to control group(s) regarding the outcome measured;—negative results—experimental group(s) did not progress or become worse in comparison with control group(s).

## Data Availability

The data that support the findings of this study are included in this article. Further inquiries can be directed to the corresponding author.
